# The association between infant non-nutritive suck and oral motor development

**DOI:** 10.1016/j.infbeh.2024.101993

**Published:** 2024-09-19

**Authors:** Ross Westemeyer, Morgan Hines, Alaina Martens, Emily Zimmerman

**Affiliations:** a Department of Communication Sciences and Disorders, University of Northern Iowa, Cedar Falls, IA, USA; b Department of Communication Sciences and Disorders, Northeastern University, Boston, MA, USA

**Keywords:** Infant, Non-nutritive suck, Oral motor skills, Motor development

## Abstract

This study investigated if non-nutritive suck (NNS) at 3 months is related to subsequent oral motor and motor skills using caregiver-reported scores on the Child Oral and Motor Proficiency Scale (ChOMPS) at 12 months in a cohort of 69 full-term infants and their caregivers. Longer NNS burst durations were associated with lower oral motor coordination and total ChOMPS scores. More NNS cycles per minute was associated with lower complex motor movement scores. More NNS bursts, cycles per burst, and cycles per minute were related with lower total ChOMPS scores. Early NNS outcomes can provide valuable insight in future neuromotor development.

## 1. Introduction

Non-nutritive suck (NNS) refers to infant sucking performance without nutritional intake (e.g., sucking on a pacifier, thumb, or empty breast). This oral sensorimotor behavior is characterized by a series of rhythmic compression/suction cycles of the jaw and tongue (burst) separated by pauses for breathing. NNS can be observed in the womb as early as 15 weeks’ gestational age (GA; Humphrey, 1970; Miller et al., 2003) and is coordinated by 34 weeks’ GA (Hack et al., 1985). Typical NNS patterning was described in 26 full-term infants at 3 months of age and these data revealed a median of 4.50 suck bursts per minute with 9.60 cycles per burst at a median frequency of 2.09 Hz and average amplitude of 14.05 cmH2O (Martens et al., 2020). The suck central pattern generator in the brainstem is the primary neural basis of NNS and is adaptable by numerous afferent and efferent pathways to modify its performance in response to sensory stimuli (Poore et al., 2008; Zimmerman & Foran, 2017). Specifically, patterned auditory (Zimmerman & Foran, 2017), visual (Zimmerman & DeSousa, 2018), and orocutaneous somatosensory (Poore et al., 2008) stimulation have been reported to influence NNS parameters. NNS can provide insight into future feeding performance, as more stable NNS measures have been associated with subsequent feeding success and a shorter transition to full oral feeds in premature infants (Bingham et al., 2010; Pineda et al., 2019).

The complex neural representation of NNS and its close association with feeding outcomes make NNS a valuable indicator of current neuromotor status and future neurodevelopment. Nutritive and non-nutritive sucking behavior in premature infants assessed by the Neonatal Oral-Motor Assessment Scale (NOMAS; Palmer et al., 1993) between 37 and 50 weeks’ post-conceptual age was significantly related with neurodevelopmental outcomes at six months (Zhang et al., 2017) and two years of age (Wolthuis-Stigter et al., 2015), and motor, intelligence, and language outcomes at five years of age (Wolthuis-Stigter et al., 2017). Specifically, poor sucking performance was associated with abnormal neurodevelopmental scores at six months and two years and better sucking skills were related with higher scores in several developmental domains at five years (Wolthuis-Stigter et al., 2015; Wolthuis-Stigter et al., 2017; Zhang et al., 2017). Retrospective studies have also reported similar findings, as children with a history of feeding difficulties had higher risks for subsequent language, cognitive, and motor impairments (Adams-Chapman et al., 2013; Malas et al., 2015). Further, a recent study reported that several NNS parameters measured at 3 months had statistically significant negative relationships with cognitive domain and general scores in the Development Profile 3 (DP-3) at 12 months (Martens et al., 2024). Although early sucking performance has been associated with future neurodevelopment, there is a paucity of research on its relationship with skilled oral motor development.

Oral motor skills refer to coordinated performance of oral structures to complete tasks like sucking, biting, and chewing (Sanchez & Morgan, 2018). The evolvement of oral motor skills is intertwined with brain development, feeding and communication experience, and a growing oropharyngeal anatomy (Bahr, 2015). Compared to healthy infant cohorts, disorganized NNS behavior has been observed in several clinical populations including premature infants (Capilouto et al., 2014) and infants with neurologic disorders (Hafström & Kjellmer, 2001). Interestingly, these clinical populations have been reported to demonstrate oral motor dysfunction within the first year of life (Buswell et al., 2009; Reilly et al., 1996). Since NNS is one of the first occurring motor behaviors in infants, it could be a foundational motor skill in which further feeding developmental processes are built upon (Poore & Barlow, 2009). Therefore, this study investigated if NNS at 3 months is related to oral motor skills at 12 months. We hypothesized that longer burst duration and more bursts, suck cycles per burst, and suck cycles per minute and lower NNS amplitudes at 3 months of age would be related with lower, or worse, caregiver reported scores of motor development, specifically oral motor skills, at 12 months of age in full-term infants. These hypotheses are based on prior work from Martens et al. (2024) who reported that longer NNS bursts with more suck cycles per burst and suck cycles per minute at 3 months were associated with lower cognitive scores in the DP-3 at 12 months.

## 2. Methods

The study was approved by the authors’ institutional review board on August 19th, 2019. The present study used a prospective longitudinal research design and was part of a larger project examining the interplay between oral motor, communication, and neurodevelopment in the first few years of life in preterm and full-term infants.

### 2.1. Participants

Infants and their caregivers were recruited using various efforts including social media posts and word of mouth about the study. Infants were excluded from study participation if they were born preterm (born < 37 weeks’ GA) or had any caregiver-reported history of neurologic or chromosomal disorders. Caregivers provided informed consent for themselves and their infants for study participation. This exploratory study on 69 caregiver-infant pairs utilized a convenience sample from a larger ongoing study. The sample size in this study is greater than other studies that reported statistically significant associations between NNS variables and measures of future neurodevelopment similar to this project (Martens et al., 2024; Murray et al., 2021). Caregiver and infant demographics are presented in Table 1.

### 2.2. Measures

**Fig. 1.**
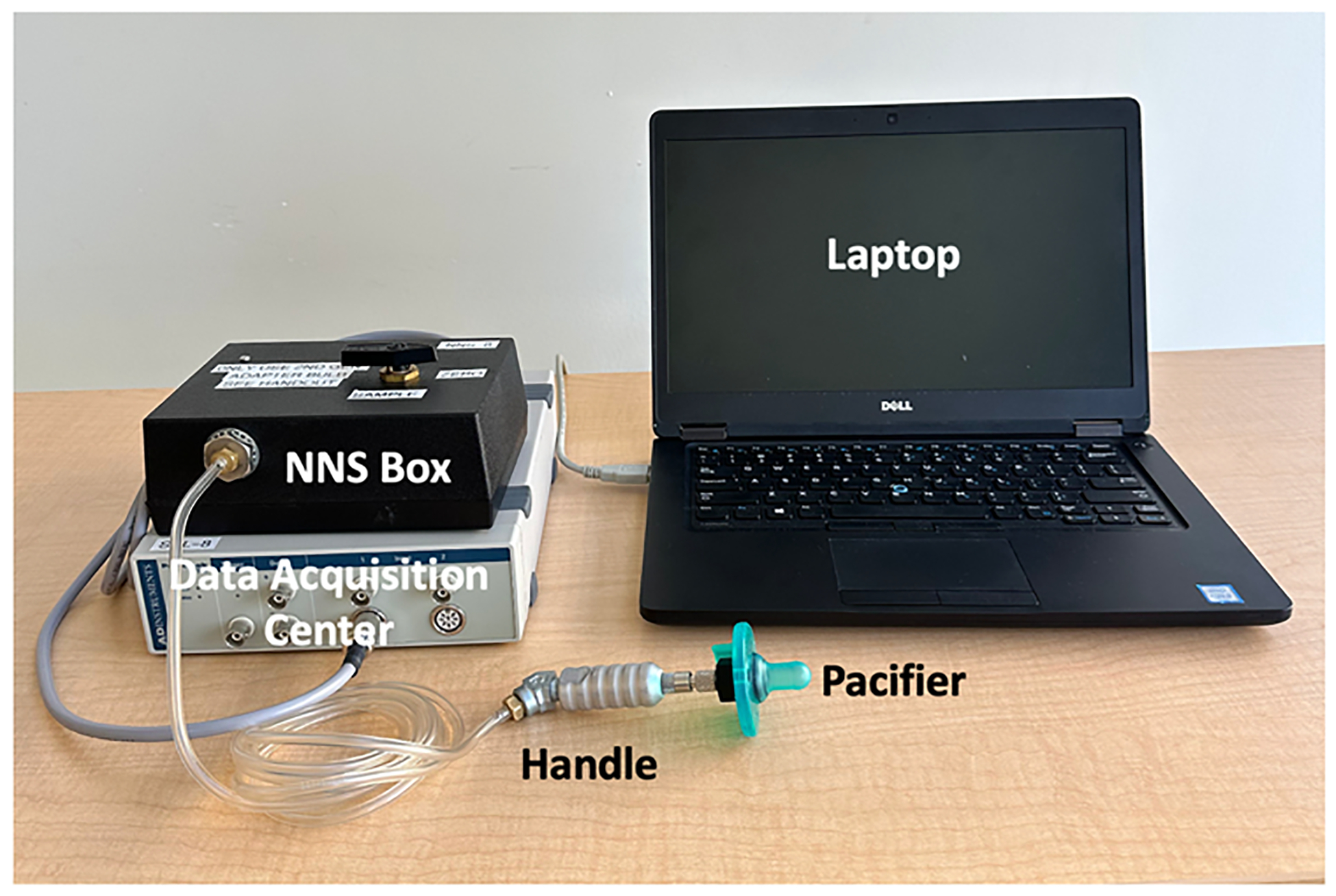
NNS System. *Note*. Device components are labeled. The portable system includes a pacifier attached to the end of a handle that is connected to a customized pressure transducer (NNS Box) that is connected to a data acquisition center (PowerLab). The data acquisition center connects to a laptop with LabChart software that records NNS data.

NNS was assessed by a trained researcher at the infant’s home approximately one hour before a scheduled feed. NNS data were collected using a Soothie pacifier (Phillips Avent, Amsterdam, Netherlands) connected to a custom pressure transducer system which was recorded via a PowerLab data acquisition device and LabChart software (ADInstruments, Dunedin, New Zealand; Westemeyer et al., 2024). The NNS system (Fig. 1) was calibrated to a ground truth signal prior to each data collection session, ensuring the NNS pressure measurements were accurate. A caregiver held and offered their infant the research pacifier and NNS behavior was collected for 2–5 consecutive minutes (Zimmerman, Carpenito, et al., 2020). If infants fell asleep or displayed signs of discomfort during NNS sampling, researchers would discontinue data collection.

After data collection visits, NNS analysis was completed by trained researchers using LabChart software. NNS bursts with 2 or more suck cycles that had an amplitude greater than 1 cmH2O and were within 1000 ms of each other were manually selected for analysis. These NNS bursts were then entered in a custom NNS Burst Macro that provided the following NNS outcome variables: burst duration (s), frequency (Hz), amplitude (cmH2O), bursts (amount of bursts that occur in a minute), cycles per burst (number of NNS cycles within a burst), and cycles per minute (amount of cycles that occur in a minute). The highest NNS cycle count sampled across 2 consecutive minutes was used to calculate minute rate averages.

Caregivers completed the Child Oral and Motor Proficiency Scale (ChOMPS; Pados et al., 2019) when their infants were 12 months of age. Completion of ChOMPS took approximately 15 min for caregivers. ChOMPS is a valid and reliable caregiver-reported assessment that evaluates feeding, oral motor, and other motor skill development in children between the ages of 6 months and 7 years (Park et al., 2019). Caregivers responded to 63 motor-related items using a Likert scale with options of “YES,” “SOMETIMES,” AND “NO” to describe if their child performs motor tasks across several developmental domains.

ChOMPS outcomes include a total score and scores in the following subscales: complex movement patterns, basic movement patterns, oral motor coordination, and fundamental oral motor skills. The complex movement patterns subscale includes questions on gross motor performance (i.e., standing, walking, jumping) and broad feeding skills (i.e., cup drinking, utensil use, lingual function, mastication). The basic movement patterns subscale was comprised of questions on a child’s abilities in gross and fine motor skills (i.e., crawling, rolling over, grasping/holding/manipulating objects, bringing hands to mouth) and postural control (i.e., sitting, supporting their head). The ChOMPS subscale of oral motor coordination asks questions on a child’s oral motor performance during feeding and abilities to accept and eat a variety of textures without gagging, coughing, or choking. Lastly, the fundamental oral motor skills subscale evaluates a child’s skills in a few basic oral motor movements (i.e., closing lips, protruding and lateralizing the tongue, opening and closing the jaw). Higher ChOMPS scores indicate more motor skills per caregiver report.

### 2.3. Statistical Analysis

Statistical analyses were performed using IBM SPSS Statistics v.28 (Armonk, New York, USA) and GraphPad Prism 10 (Boston, Massachusetts, USA). A correlation matrix among NNS and ChOMPS variables was completed to investigate the statistical relationship across study outcomes. Shapiro Wilk tests indicated NNS and ChOMPS data were non-normally distributed, so Spearman correlations were then applied to the strongest correlations to investigate the bivariate relationships between NNS outcomes and ChOMPS scores. Alpha level significance was set at *p* < 0.05 for Spearman correlations. Missing data (all NNS variables for one participant, one ChOMPS sub-scale and total score for two participants, and two ChOMPS subscales and total scores for one participant) were excluded from analysis.

## 3. Results

**Fig. 2.**
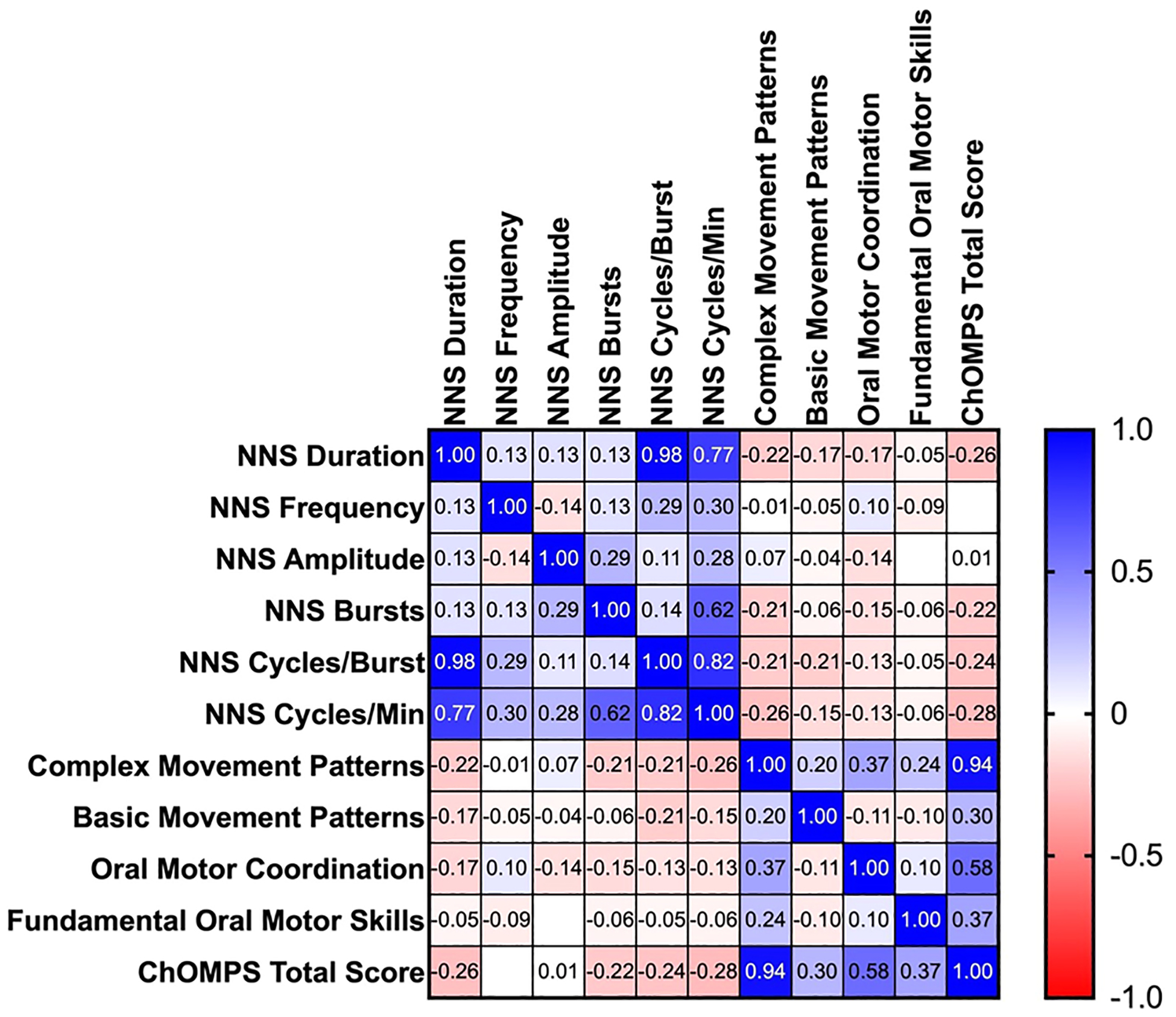
Correlation Matrix among NNS and ChOMPS Outcomes. *Note*. Darker shades indicate higher correlations among variables and lighter shades indicate weaker correlations.

The correlation matrix of the relationships between NNS outcomes at 3 months of age and ChOMPS scores at 12 months of age can be seen in Fig. 2. From Fig. 2, we completed Spearman correlations on any correlation above 0.15 and this revealed several statistically significant relationships. Longer NNS burst durations were related with lower scores on the ChOMPS oral motor coordination subscale (*r* = −0.28, *p* = 0.021) and total score (*r* = −0.31, *p* = 0.011). More NNS bursts (*r* = −0.26, *p* = 0.036) and cycles per burst (*r* = −0.30, *p* = 0.016) were associated with lower ChOMPS total scores. Lastly, more NNS cycles per minute was related with lower scores in the complex movement patterns subscale (*r* = −0.24, *p* = 0.048) and ChOMPS total score (*r* = −0.31, *p* = 0.012).

## 4. Discussion

This study investigated if NNS at 3 months was related with subsequent oral motor skills at 12 months. There were several significant associations between NNS variables and ChOMPS scores. In support of the research hypothesis, longer burst durations and more bursts, cycles per minute, and cycles per burst were related with lower scores in ChOMPS subscales and total score. Specifically, these NNS parameters were associated with lower caregiver reports of skilled motor acquisition in fine, gross, and feeding-related movements. Longer NNS burst durations were associated with lower oral motor coordination subscale scores, which indicates limited oral motor performance in accepting various textures without gagging, coughing, or choking. In contrast to the research hypothesis, amplitude measures were not significantly related to any ChOMPS subscales or total scores.

Results from this study are largely consistent with prior research that has investigated NNS and future motor, cognitive and communication-related performance. An interpretation of normal sucking pattern by 50 weeks’ postmenstrual age, measured by the NOMAS, in a cohort of premature infants was associated with higher skilled motor performance scores in manual dexterity, ball skills and balance tasks at five years (Wolthuis-Stigter et al., 2017). In a recent research study from Martens et al. (2024), longer NNS burst durations and more cycles per burst and cycles per minute at 3 months were associated with lower cognitive development scores at 12 months in full-term infants. Additionally, Murray et al. (2021) reported longer burst durations and higher intraburst frequency of NNS data sampled at 3 months was predictive of increased variability of voice onset time at 12 months. This pattern of more suck cycles per burst being associated with poorer cognitive and more variable communication outcomes could be partially explained from what is understood about NNS development. NNS parameters besides frequency evolve during the first year of life (Martens et al., 2020), most likely attributed to neural maturation, growth of the oropharyngeal structures, and feeding and communication experiences (Bahr, 2015). Specifically, NNS bursts per minute, cycles per burst, and burst duration decrease with age while NNS amplitude increases, which indicate these NNS outcomes are representative of a more mature and organized suck performance (Martens et al., 2020).

Contrary to our hypothesis, NNS amplitude was not related with caregiver-reported oral motor performance. Higher NNS amplitudes have been observed in a small cohort of full-term infants compared with preterm infants, although this effect did not reach statistical significance (Capilouto et al., 2014). Greater measures of NNS amplitudes could represent stronger and more coordinated lingual function, which would theoretically be favorable for oral motor development as the tongue is a pivotal structure in numerous feeding-related milestones (Arvedson et al., 2019). Larger amplitudes during sucking behavior could also be indicative of mature motor patterns, as Lau et al. (2000) observed amplitude measures increasing during stages of sucking development in premature infants which was associated with more efficient feeding. Although NNS amplitude was not related to ChOMPS outcomes in this study, its relationship with future neural and motor development should continue to be explored, particularly in clinical populations.

Results from this study have meaningful clinical implications that could facilitate oral motor developmental trajectories in infants and help identify those at-risk for atypical oral motor development. Although NNS is not directly indicative of nutritive sucking success because safe and efficient feeding requires mature physiologic systems and skilled coordination between sucking, swallowing, and respiration (Lau, 2016), its performance undoubtedly provides insight into the sucking motor behavior essential in the suck-swallow-breathe feeding pattern. This is supported by the research literature of investigations that use NNS as a pro-habilitation strategy for feeding development and outcomes in clinical populations. A meta-analysis of 12 studies investigating the effects of NNS practice in premature infants reported significant effects of quicker transitions from gavage feeds and initiation of oral feeds to full oral feeding and shorter lengths of hospital stay compared to premature infants who did not receive NNS intervention (Foster et al., 2016). Furthermore, NNS performance can facilitate secretion of gut hormones to support digestion (Marchini et al., 1987) and has been reported to alleviate pain and maintain an optimal behavioral state in premature infants (Bingham et al., 2011; Liaw et al., 2012). These effects of early NNS practice could prime positive feeding performance, which is a primary factor that drives feeding success (Pickler et al., 2006) and consequent development of oral motor skills (Lau, 2016).

The study’s findings suggest that longer burst duration and more bursts, cycles per burst, and cycles per minute in NNS performance could be indicative of immature oral sensorimotor patterns. Observation of these NNS parameters could help identify infants who have a greater risk for delayed or dysfunctional oral motor development. These results have valuable prognostic implications and could provide insight into current neuromotor status and future trajectories in oral motor, feeding, and communication development. Identification of at-risk infants using a sensitive measure of neurodevelopment like quantitative assessment of NNS could lead to earlier access to intervention services.

This study has several limitations that are essential to acknowledge. The significant Spearman correlations in this study ranged between −0.26 – −0.31, which are weak to moderate. Further, the external validity of results is limited due to the homogenous cohort of infants and caregivers. What we found compelling in this study was the consistent association between NNS and ChOMPS measures that warrant further study with a larger sample size. Another limitation was that ChOMPS scoring is reliant upon caregiver report and were not direct physiologic measures of feeding-related motor performance. An important next step after this study is to investigate the relationships between early NNS and future oral motor development in larger, more diverse samples to improve generalization and interpretation of results. Further research in oral motor development, particularly in clinical populations, is needed to characterize oral motor skills as disordered or delayed. A better understanding of aberrant oral motor development has immense value, especially in consideration of the substantial variation in “typical” motor skill acquisition and mastery in the first two years of life that has been observed in large, homogeneous cohorts (Carruth & Skinner, 2002). Additionally, future studies could benefit from more direct and precise physiologic measurements of oral motor skills and function (i.e., performance of nutritive sucking, mastication of solid foods, spoon feeds, open-cup drinking).

The multiple statistically significant relationships between early NNS behavior and caregiver-reported scores in oral motor-centric skilled acquisition support the idea of NNS being a foundational skill that may contribute to the development of other oral sensorimotor behaviors (Poore & Barlow, 2009); however, the development of oral motor and feeding skills should be acknowledged as multi-determined. Scarborough et al. (2018) highlights the complexities of achieving just one feeding milestone of open cup drinking and how its success is contingent upon the culmination of development in oral motor, feeding, fine motor, postural, and cognitive skills. Zimmerman, Carnaby, et al. (2020) further underscores the dynamism of feeding development by using Thelen’s (1985) Dynamic Systems Theory framework to discuss the numerous factors between the child, task and environment that impact feeding performance. The study’s results of NNS being intertwined with the acquisition of subsequent oral motor skills emphasize the major future direction of studies investigating the underlying mechanisms that link NNS with a broad range of neurodevelopmental outcomes. A better understanding of what downstream effects NNS may have on future oral motor, feeding, communication, and cognitive performance can illuminate early intervention approaches to help get children on optimal neurodevelopmental trajectories.

Infant NNS measured at 3 months of age had several statistically significant relationships with caregiver-reported motor outcomes at 12 months in a group of full-term infants. Longer NNS durations and more NNS bursts, cycles per minute and cycles per burst were associated with lower reports of skilled motor acquisition in complex motor movement and oral motor coordination domains. Further research is warranted to elucidate the mechanisms that connect early NNS parameters with future oral motor performance. These results highlight that early and quantifiable NNS assessment has utility as a sensitive biomarker of subsequent neurodevelopment.

## Figures and Tables

**Table 1:** Infant and Caregiver Demographics.

	Infant Demographics (N = 69)	
Male	38 (55 %)	
Birthweight (SD)	7.58 (0.99) pounds	
Gestational Age (SD)	39.28 (1.19) weeks	
Age at 3-month session (SD)	2.99 (0.27) months	
Age at 12-month session (SD)	11.99 (0.33) months	
	Caregiver Demographics (N = 69)	
Age at 3-month session (SD)	34.31 (3.64) years	
Married	65 (97 %)	
Education	College degree or higher	62 (90 %)
	Master’s degree or higher	51 (74 %)
Race	White	55 (80 %)
	Asian	10 (15 %)
	Black	1 (1 %)
	Hispanic/Latino	1 (1 %)
	Preferred not to label	1 (1 %)
	Unreported	1 (1 %)

## Data Availability

Data will be made available on request.

## Abbreviations:

NNS: non-nutritive suck
ChOMPS: Child Oral and Motor Proficiency Scale
GA: gestational age
NOMAS: Neonatal Oral Motor Assessment Scale
DP-3: Development Profile 3
